# Importance of water level management for peatland outflow water quality in the face of climate change and drought

**DOI:** 10.1007/s11356-022-20614-2

**Published:** 2022-06-02

**Authors:** Shokoufeh Salimi, Miklas Scholz

**Affiliations:** 1 Division of Water Resources Engineering, Faculty of Engineering, Lund University, P.O. Box 118, 221 00 Lund, Sweden; 2 School of Science, Engineering and Environment, The University of Salford, Newton Building, M5 4WT Salford, United Kingdom; 3 Department of Civil Engineering Science, School of Civil Engineering and the Built Environment, University of Johannesburg, Kingsway Campus, Aukland Park 2006, PO Box 524, Johannesburg, South Africa; 4 Department of Town Planning, Engineering Networks and Systems, South Ural State University (National Research University), Prospekt Lenin 76, Chelyabinsk, 454080 Russia

**Keywords:** Bog, Global warming, Representative concentration pathway, Peat decomposition, Nutrient release, Dissolved organic carbon, Nitrogen, Phosphorus

## Abstract

The impact of different climate scenarios, drought, and water level management on the outflow water quality of peatlands has been investigated. A mesocosm experiment has been conducted within climate control chambers to simulate current (2016–2019 real-time) and future representative concentration pathway (RCP) climate scenarios (RCP 2.6, 4.5 and 8.5). To assess the efficiency of a management strategy for improving peatland water quality, water level adjustment was applied to half of the system at the same time for each climate scenario. Furthermore, the mesocosm experienced the 2018 European drought during the simulation years, and the corresponding impact was analyzed. The results of this study revealed a substantial and favorable impact of water level management on water quality of peatlands under different climate scenarios. The effect of water level management was the largest for ammonium (NH_4_-N) and 5-day biochemical oxygen demand (BOD_5_), and the smallest for total phosphorus (TP). Drought had a strong impact on chemical variables, increasing their concentration and deteriorating the water quality of peatland outflow. However, water level management can stabilize the nutrient levels in peatland outflows, particularly during drought and under warmer climate scenarios, thus mitigating the adverse effects of climate change.

**Supplementary Information:**

The online version contains supplementary material available at 10.1007/s11356-022-20614-2.

## Introduction

Peatlands are unique ecosystems that cover around 3% of the global land surface and are one of the most vulnerable ecosystems to climate change (Harenda et al. 2018). Peatlands, like other wetland ecosystems, serve as a transitional zone where terrestrial and aquatic environments collide. Therefore, any kind of peatland disturbance such as a collapse of parts of the ecosystem has an impact on the aquatic ecosystem. Peatlands can be considered as storage systems of carbon in the landscape (Bragazza et al. 2013). They have the ability to accumulate dead organic matter (transformed into peat over time) when the rate of plant litter generation exceeds the rate of peat degradation, which occurs under prevailing waterlogging conditions (Limpens et al. 2008). One of the most unique and valuable services of a pristine peatland is retention of carbon and nutrients and prevention of erosion, delivering improved water quality to downstream regions (Ritson et al. 2016; Tuukkanen et al. 2017).

The high moisture retention capacity of a peatland is regarded as a crucial regulator of this ecosystem increasing its resilience to any disturbances (Waddington et al. 2015). Climate events such as droughts are expected to become more common as a result of climate change–related alterations in temperature and precipitation (Erwin 2009; Salimi et al. 2021a). This might have direct and indirect consequences on peatland functionality in the catchment. Less water availability as a result of a higher rate of evapotranspiration under climate change and drought might cause an aerobic decomposition of peat and release of stored nutrients and pollution into the peatland outflow, harming the downstream water quality (Macrae et al. 2013; Juckers and Watmough 2014; Tuukkanen et al. 2017). Moreover, factors that contribute to peatland degradation, such as severe droughts, not only accelerate the rate of organic matter decomposition but also increase the amount of carbon dioxide (CO_2_) emission into the atmosphere (Lucchese et al., 2010; Grover and Baldock 2013; Jassey et al. 2018). The high moisture retention capacity of peatlands is recognized as a critical regulator of peatland functions. Nonetheless, studies revealed that optimal ecosystem services of peatlands depend on careful water level management, keeping the water level within specified limits to prevent them from becoming overly wet or too dry (Macrae et al. 2013; Menberu et al. 2017). This is critical for preserving peatland ecosystem services including flood and nutrient retention, carbon sequestration, and biodiversity (Salimi et al. 2021a).

The complexity of the response of the peatland ecosystem might be attributed to some degree to the fact that the vegetation composition would shift gradually as a result of climate change, and this shift may modify nutrient demands and potential uptake by the vegetation (Turetsky 2003). As a result, while the warmer climate and lower water level would promote peat degradation and nutrient release (Belyea and Malmer 2004; Strack et al. 2008; Macrae et al. 2013), the altered plant composition could increase nutrient uptake and offset the adverse implications of warmer climate on peatland ecosystems to some extent (Salimi and Scholz 2021). However, other studies have found that the rate of nutrient uptake by plants may be slower than the rate of organic matter mineralization, raising concerns about net nutrient leaching from peatlands in the face of warmer climate (Westman and Laiho 2003).

Providing a stable hydrologic regime for the peatland supports the dominance of the key plant *Sphagnum* moss, which protects the bog from being readily degraded, retaining the carbon storage of the peatland. In contrast, higher water table fluctuations as a result of climate change are anticipated to change the plant and microbial community affecting nutrient mineralization patterns and carbon cycling. Predicting the extent and direction of the response of peatland water quality to climate change and drought is challenging (Moore et al. 1998; Li et al. 2020). Since the literature contains contradictory results regarding the impact of water table changes on nutrient release (Tiemeyer et al. 2007; Urbanová et al. 2011; Macrae et al. 2013), understanding the water level management effect on water quality is critical (Salimi and Scholz 2021). Moreover, as various climate scenarios might demand different types of management including water level adjustment, the assessment of different management strategies for different climate scenarios is therefore necessary.

A mesocosm experiment started in 2017 and first focused on the impact of different climate scenarios on the water quality of constructed wetlands and peatlands (ombrotrophic bog) while the water level was regulated for all mesocosms (four replicates) over the period from 2017 to 2018 (Salimi and Scholz 2021). Since 2018, the authors started to investigate the impact of both climate change and water level management and their interactions on water quality and carbon dioxide sink function of peatlands (Salimi et al. 2021b) and divided the mesocosms into two groups of managed and unmanaged (water level management) treatment (two replicates each). This study is unique in terms that it is the first time a dynamic simulation of current and future RCP climate scenarios has been conducted within climate control chambers for peatlands allowing for a close understanding of changes in the peatland ecosystem. Furthermore, water level management was undertaken concurrently to understand the efficiency of this management action for all simulated climate scenarios (Salimi et al. 2021b). The simulated climate scenarios were based on real-time data for 2017, 2018, and 2019. Although 2017 was considered a year with regular rain during the warm season, 2018 was recorded as the warmest and driest summer since 1950, and 2019 had the second warmest summer since 1950, but fairly typical precipitation (Fig. S2b,d).

The main objectives of this research were to (a) assess the impact of different climate scenarios on peatland water quality (Salimi and Scholz (2021) explored this objective for peatlands and constructed wetlands, when the water level was regulated.); (b) assess the effect of drought on peatland degradation; and (c) evaluate the impact of water level management on peatland water quality under different climate scenarios (Salimi et al. (2021b) investigated the effect of water level management on the carbon dioxide sink function of peatlands under various climate scenarios.).

## Materials and methods

### Climate scenario creations for subsequent simulations

In this study, the scenarios called representative concentration pathway (RCP) were used for creating future climate scenarios for the climate chambers. These RCP future climate scenarios are based on different radiative forcing target levels for the future and were provided by the Intergovernmental Panel on Climate Change (IPCC) (AR5; United Nation 2014). According to the IPCC’s Fifth Assessment Report (AR5; United Nation 2014), there are four possible scenarios: RCP 2.6, RCP 4.5, RCP 6, and RCP 8.5 (Van Vuuren et al. 2011). Three RCP climate scenarios (RCP 2.6, RCP 4.5, and RCP 8.5) were used to simulate low, moderate, and extreme future climate change scenarios in the corresponding climate chambers.

Table S1 lists the regional climate change models (RCMs), which have been used for the simulation of future scenarios. Hourly data of the different climate variables temperature, precipitation, relative humidity, and radiation for the regional climate models (RCM) for various RCP scenarios were collected from the Rossby Centre of the Swedish Meteorological and Hydrological Institute (SMHI). There were five RCM datasets available at SMHI for RCP 4.5 and 8.5 and 10 RCM datasets for RCP 2.6, 4.5, and 8.5. The data were collected for the domain of Scania County located in southern Sweden.

Since all RCM outputs are uncertain, no single RCM was used to create the climate scenarios, instead, the delta change technique was used to include all existing RCMs to reduce uncertainty. In this method, the difference between the output of each RCM for the last 30 years of the century (2069–2098) and the historical data of the same models for the same number of years (1976–2005) has been estimated. The results were then averaged across all RCM models for all RCPs, and, subsequently, the estimated differences (for temperature) and ratios (for precipitation, relative humidity, and radiation) were calculated for each month resulting in monthly delta change coefficients. The calculated monthly delta change coefficients were applied to the hourly observation data of Malmö and Lund station for the years 2016–2019 (current climate) to generate future climate scenario values. Finally, 3-h values for temperature, relative humidity, and radiation for all climate scenarios were computed and used for the climate chambers to simulate the current and future climate change scenarios.

### Collection of peat turfs

Peatland samples were extracted from Fäjemyr, which is an ombrotrophic bog in the province of Scania (latitude 5615′N, longitude 1333′E and altitude of 140 m). The samples were collected from the top layer of the peatland and deposited directly in tanks (30 cm in length, 22 cm in width, and 24 cm in height) during fieldwork. The peatland mesocosms are made-up of 20 cm of top bog plants with some young peat at the bottom. Fäjemyr vegetation is dominated by dwarf shrubs (*Calluna vulgaris* and *Erica tetralix*), sedges (*Eriophorum vaginatum*), and *Sphagnum* spp. (*S. magellanicum* and *S. rubellum*) (Lund et al. 2012). All mesocosms were built to be indicative of the site, with relatively similar proportions of dominant plant types.

### Advanced climate chambers

**Fig. 1:**
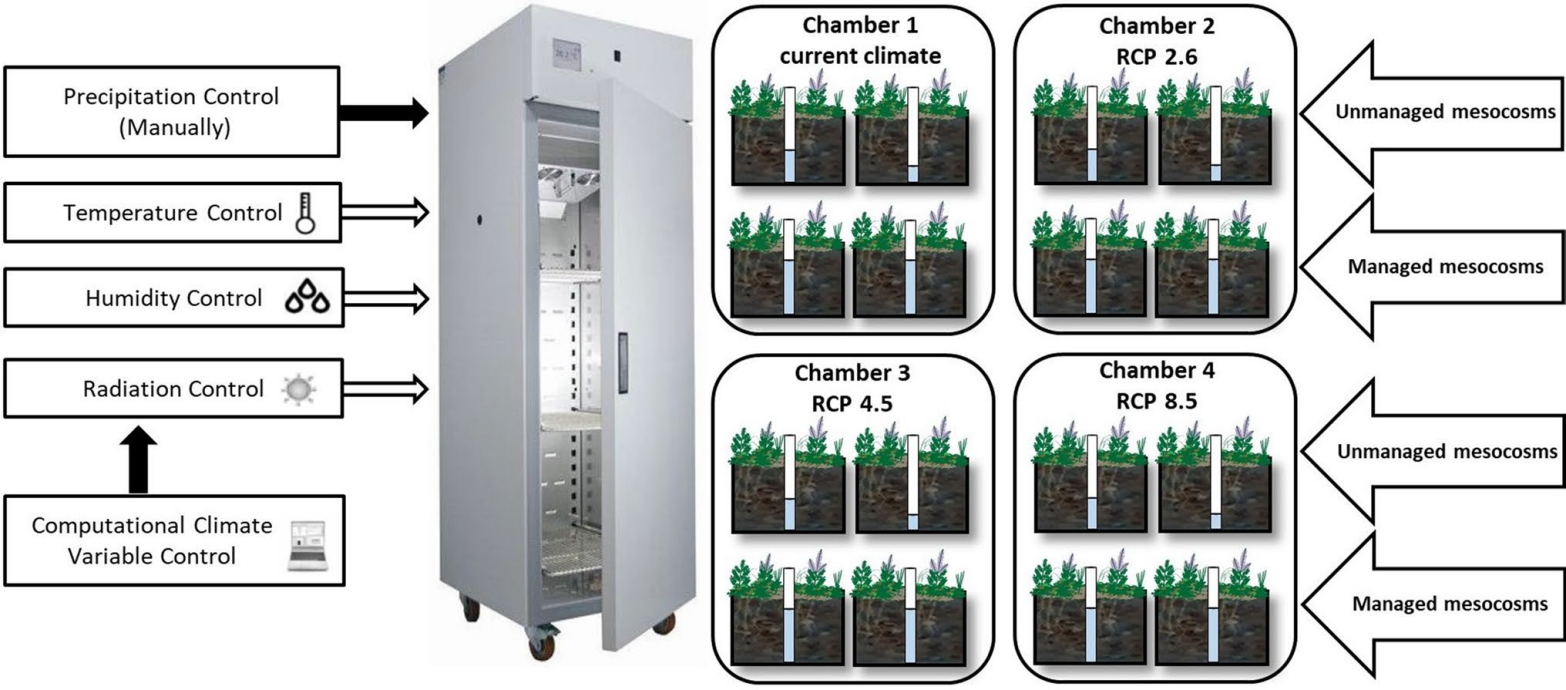
Design of mesocosm experiment in climate control chambers simulating the current and future representative concentration pathway (RCP) climate scenarios and water level management for peatlands during 2018–2020. During the first year of the experiment, all four peatland mesocosms were subject to water level management (2017–2018). In the second and third years of the experiment (2018–2020), two mesocosms were not managed (unmanaged treatment), but another two mesocosms were continued to be managed in terms of water level (managed treatment)

The authors employed four advanced climate chambers KK 750 (Pol-Eko-Aparatura Wodzisaw Slski, Poland; https://www.poleko.com.pl/model/climatic-chambers-kk/climatic-chamber-kk-750/) to simulate the climate scenarios. The climate chambers are remotely programmable allowing the user to regulate the temperature and relative humidity, as well as radiation at a resolution of 3 h (Fig. 1). However, precipitation simulation was conducted manually on a weekly basis. To ensure a realistic and dynamic simulation, all four climatic variables were simulated continuously throughout the year, with no gaps during the cold season (Salimi et al. 2021b).

The climate chambers are equipped with a phytotron system to regulate temperature and humidity. Moreover, to simulate day and night, each climate chamber is supplied with an 840 (daylight) fluorescent lamp. The climate chamber is able to control the intensity and duration of the illumination. The chamber regulates the temperature from − 10 to + 60 °C with the light switched-off, and from 0 to + 50 °C with the light on. Each climate chamber has an ultrasonic humidifier that has been connected to the deionized water system to provide the chamber with the desired humidity level. An air flap and a ventilator were also installed to ensure that air extraction and circulation are consistent.

### Design of the mesocosm experiment

The simulation of four different climate scenarios (the current climate scenario and three future RCP climate scenarios) was carried out in four advanced climate chambers as follows: chamber 1 (current climate), chamber 2 (RCP 2.6), chamber 3 (RCP 4.5), and chamber 4 (RCP 8.5). Sixteen peatland mesocosms were randomly distributed into four climate chambers, each comprising four peatland mesocosms (Fig. 1). These four mesocosms were divided into managed and unmanaged treatment (two mesocosms for each treatment) to understand how changes in water level might affect the water quality of peatland outflow under different climate scenarios (Fig. 1). The water levels for the managed treatment were regulated, but not in the unmanaged treatment (see “Water level management”).

### Water level management

From 1 October 2017 to 30 September 2018, which is considered to be a hydrologic year in Sweden, the water level in all four peatland mesocosms was regulated (Salimi and Scholz 2021). Since 1 October 2018, the four peatland mesocosms in each climate chamber have been divided into managed and unmanaged treatment (each two mesocosms) to assess the impact of water level management. Precipitation was simulated weekly for all mesocosms in all chambers using precipitation data from the generated climate scenarios explained above. Rainwater was collected from nearby greenhouse glass roofs and manually applied to the mesocosm top surface, where it infiltrates into the peatland soil, and eventually is released into the standing pipe located in the center of each mesocosm. The water level in the mesocosms was regulated for two managed mesocosms to be comparable to the unmanaged ones in terms of water quality.

After monitoring the mesocosm hydrology during the experimental set-up period, the authors noted that water levels higher than 18 cm might pose a risk of flooding (overflow of inflow) and thus loss of nutrients from the mesocosms, which might be important for plant photosynthesis. Furthermore, water levels lower than 10 cm might be insufficient to meet the water demand of *Sphagnum* spp. photosynthesis. Therefore, the water levels between 10 (± 0.5) and 18 (± 0.5) cm (from the bottom of the tank) were considered as acceptable thresholds for mesocosms to provide all plants with water and nutrients for photosynthesis. Water level management was accomplished either by adding water from the nearby real storm water pond called Lake Lake (Sjön Sjön in Swedish), which is located on the campus of the Faculty of Engineering at Lund University, to the mesocosms when the water level dropped to less than 10 cm due to evapotranspiration or by removing excess water (runoff or management outflow) from the mesocosms when the water level exceeded 18 cm. Water was added for management purposes to the top surface of the mesocosms, while excess water was removed from the vertical standing pipe positioned in the center of each mesocosm where in practice, the mesocosm outflow would be collected via a belowground horizontal collection pipe system. Water level adjustment was not performed for the unmanaged mesocosms. Therefore, rainwater was the only inflow to the unmanaged mesocosms; as a result, some mesocosms encountered extreme events such as droughts and floods (maximum 4 cm above the topsoil).

### Physicochemical parameter measurements

The European Union’s framework Directives 1991/271/EEC established quality standards for surface fresh water, which were considered as a baseline in this study. The concentrations of many different chemical variables were monitored for the first 6 months to understand which variables would potentially exceed the water quality standards. The ranges of the chemical variables were compared to the corresponding standard values, and those variables, which were classified to be relatively close to typical regulatory threshold values, have been chosen for monitoring in this study. According to this assessment, the variables dissolved organic carbon (DOC), ammonium (NH_4_), total phosphorus (TP), chemical oxygen demand (COD), biochemical oxygen demand (BOD_5_), and pH were chosen for monitoring over a longer period of time. This study also examined physical water quality indices such as dissolved oxygen (DO) and total suspended solids (TSSs).

Dissolved organic carbon (DOC) was analyzed using the TOC analyzer TOC-V CPH-TNM-1 (Shimadzu). The BOD_5_ was measured using the OxiTop Control System OC 110® (WTW, Germany). Total phosphorus (TP) was analyzed using the inductively coupled plasma atomic emission spectrometry (ICP-OES) Optima 8300 (Perkin Elmer ELAN 6100, Perkin Elmer Inc., Waltham, Mass), in accordance with the USEPA method 200.7 (USEPA 1994). Furthermore, ammonium-nitrogen (NH_4_-N) was monitored in this study and determined by flow injection analysis (FIA) using a FIAstar 5000 Analyzer (Foss Tecator, Höganäs, Sweden) according to ISO 11732:2005 and ISO 11732, respectively. Dissolved oxygen was measured directly from the mesocosm outflow pipe with no or minimum disturbance of the mesocosms. Total suspended solids were determined using Lovibond MD 100 (VWR, Germany). The pH was measured with the pH/mV/°C meter pHenomenal® pH1100L meter (VWR, Germany).

## Statistical analyses

A two-way repeated-measures multivariate analysis of variance (RM-MANOVA) was used to assess the effects of the main factors (water level management and climate scenario) as well as their interactions on physicochemical parameter changes over time. The sampling month (time) was included as a within-subject to enable for treatment effects to be evaluated despite seasonal fluctuations of the dependent variables. Water table management and climate scenarios were considered as the between-subject factors.

To obtain a sufficient sample size in relation to the number of dependent variables, the physicochemical parameters were grouped into fewer chemical and physical parameters. The parameters with the minimal possible similarity were grouped together. Moreover, the potential autocorrelations between dependent variables were considered in the analysis. In RM-MANOVA, Wilk’s Lambda was used to determine the significance of the main factors. The RM-MANOVA effect sizes were examined using partial eta squared (p2) (Salimi and Scholz 2021). The Bonferroni adjustment test was used to determine pairwise differences. For all tests, the significance level was set at *α* = 0.05.

## Results

### Comparisons of environmental variables under different climate scenarios

Radiation showed a declining trend from the coldest (current) climatic scenario to the warmest (RCP 8.5) according to Fig. S1a. Radiation in the RCP 2.6, RCP 4.5, and RCP 8.5 scenarios had an annual average ratio of 0.98 ± 0.033, 0.95 ± 0.028, and 0.94 ± 0.033 to the current climate scenario, respectively. The RCP 4.5 and RCP 8.5 scenarios had significantly higher radiation than the current climate scenario, but not RCP 2.6. Furthermore, no significant differences in radiation were found across all future climate scenarios (Fig. S1a). A comparison of temperature means (Tukey’s test) for different climate scenarios revealed that all climate scenarios were significantly different (Tukey’s test; *p* < 0.05) from one another (Fig. S1b). From the current climate, there was an increasing trend towards RCP 8.5. The differences between the annual average temperature of the future climate scenarios RCP 2.6, RCP 4.5, and RCP 8.5 relative to the current climate (control scenario) were 1.8 ± 0.52 °C, 2 ± 0.43 °C, and 3.2 ± 0.75 °C, respectively (Fig. S1b). The differences between the relative humidity values of the scenarios were rather small: 1.003 ± 0.0113, 1.007 ± 0.0062, and 1.009 ± 0.0108 for RCP 2.6, RCP 4.5, and RCP 8.5 compared to the current climate scenario, respectively. There was no significant difference in relative humidity between the current climate scenario and RCP 2.6, or between RCP 4.5 and RCP 8.5. However, the current climate scenario and RCP 2.6 had significantly lower relative humidity values (Tukey’s test; *p* < 0.05) than RCP 4.5 and RCP 8.5 (Fig. S1c). Mean precipitation increased from the current climate scenario to RCP 8.5. The RCP 2.6, RCP 4.5, and RCP 8.5 scenarios have experienced 1.02 ± 0.082, 1.09 ± 0.072, and 1.15 ± 0.137 times higher annual average precipitation relative to the current scenarios, respectively. However, there was no significant difference among the four climate scenarios (Fig. S1d).

The water level was measured (from the bottom of each mesocosm) on a weekly basis. The averages of the water levels for the current climate, RCP 2.6, RCP 4.5, and RCP 8.5 scenarios for the unmanaged mesocosms were 10.8 ± 0.59 cm, 9.7 ± 0.60 cm, 10.4 ± 0.58 cm, and 12.3 ± 0.54 cm, correspondingly, and for the managed mesocosms, they were 12.1 ± 0.24 cm, 12.7 ± 0.22 cm, 13.1 ± 0.25 cm, and 13.0 ± 0.24 cm in this order over two years (2018–2020). There was a significant difference between the mean water level (2018–2020) of managed and unmanaged mesocosms under RCP 4.5 and RCP 8.5, but not under the current climate and RCP 8.5 (Tukey’s test; *p* < 0.05). Furthermore, the mean water level (2018–2020) of managed mesocosms was not found to be statistically different between the scenarios, with the exception of the mean water level of the managed mesocosms in the current climate, which was significantly different from RCP 4.5 and RCP 8.5 (Tukey’s test). The mean water level of unmanaged mesocosms (2018–2020) showed no significant difference between climate scenarios, with the exception of RCP 2.6, which had a significantly lower mean water level than RCP 8.5 (Tukey’s test; *p* < 0.05).

### Effect of climate scenario and water level management on physicochemical variables

**Fig. 2:**
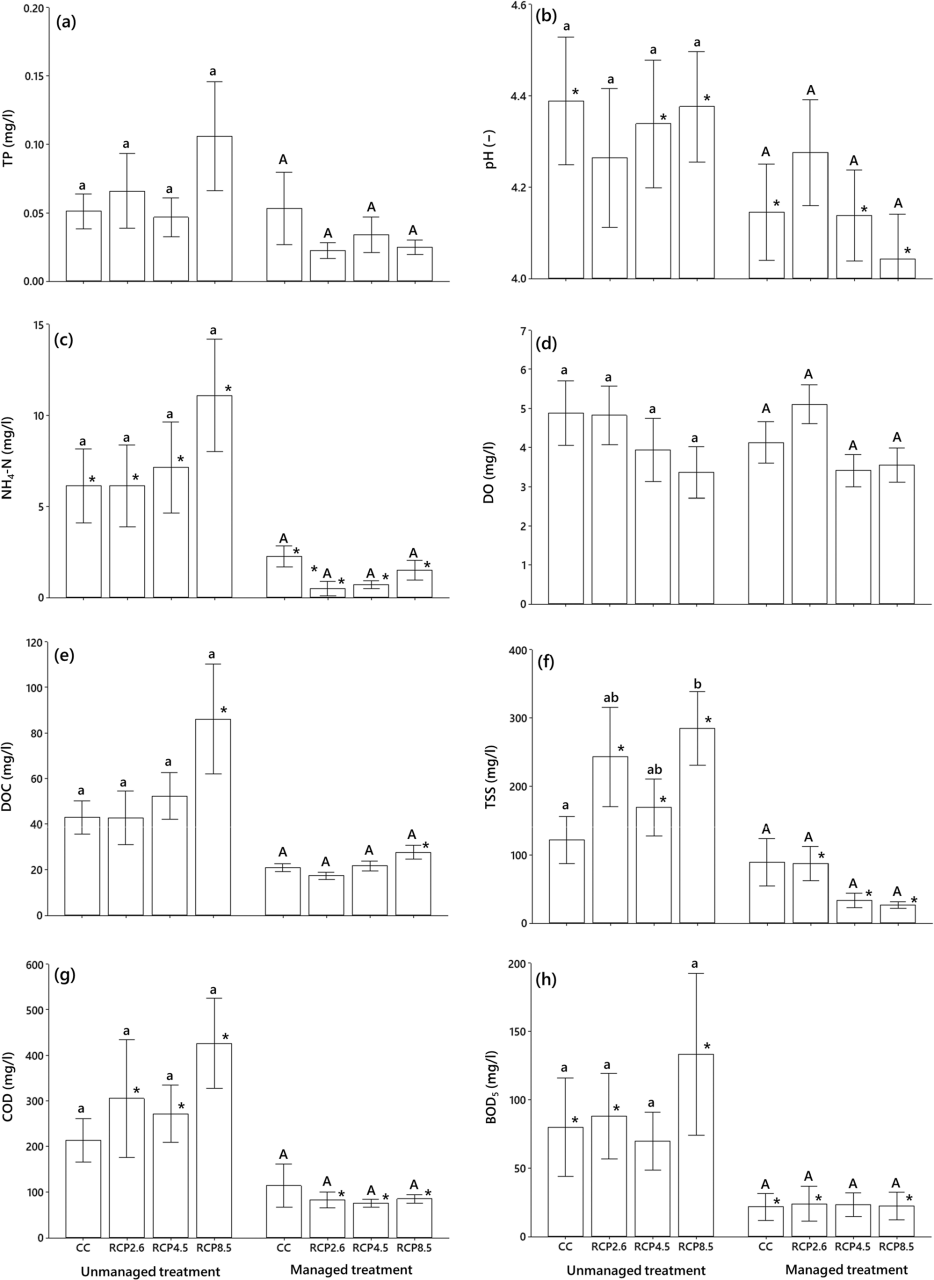
Two-year average (period 2018–2020 of the experiment simulating 2017–2019 in real time) of **a** total phosphorus (TP), **b** pH, **c** ammonium-nitrogen (NH_4_-N), **d** dissolved oxygen (DO),**e** dissolved organic carbon (DOC), **f** total suspended solids (TSS), **g** chemical oxygen demand (COD), and **h** 5-day biochemical oxygen demand (BOD_5_). Asterisks denote significant differences between the unmanaged and managed treatment for peatland mesocosms for each climate scenario. Significant differences in climate scenarios have been demonstrated using lower case letters for the unmanaged peatland treatment and upper case letters for the managed treatment. Means that share the same letter or letters are not significantly different. The significance test is based on a pairwise comparison (Bonferroni adjusted significance test) followed by a two-way repeated-measures multivariate analysis of variance (RM-MANOVA). The mean difference is significant at *α* = 0.05

Figure 2 shows the 2-year (2018–2020) averages of selected water quality variables when the mesocosms started to be compared between managed and unmanaged treatments. Salimi and Scholz (2021) have published some findings of the first year (2017–2018) when all mesocosms were subject to water level management. The significance test in this study is based on a pairwise comparison (Bonferroni adjusted significance test) followed by RM-MANOVA. The latter results revealed that the main factor (climate scenario) had no statistically significant impact on any group of variables: (a) DOC, COD, and TSS (*p* = 0.29, Wilks’ Lambda = 0.23, *η*_*p*_^2^ = 0.39); (b) NH_4_-N, TP, and BOD_5_ (*p* = 0.40, Wilks’ Lambda = 0.11, *η*_*p*_^2^ = 0.52); and (c) DO and pH (*p* = 0.17, Wilks’ Lambda = 0.21, *η*_*p*_^2^ = 0.54). However, the effect of water level management was found to be statistically significant for all groups of variables: (a) DOC, COD, and TSS (*p* = 0.002, Wilks’ Lambda = 0.10, *η*_*p*_^2^ = 0.90); (b) NH_4_-N, TP, and BOD_5_ (*p* = 0.002, Wilks’ Lambda = 0.01, *η*_*p*_^2^ = 0.99); and (c) DO and pH (*p* = 0.03, Wilks’ Lambda = 0.25, *η*_*p*_^2^ = 0.76).The interaction effect of climate scenario and water level management was not statistically significant for any groups of variables: (a) DOC, COD, and TSS (*p* = 0.33, Wilks’ Lambda = 0.25, *η*_*p*_^2^ = 0.37); (b) NH_4_-N, TP, and BOD_5_ (*p* = 0.32, Wilks’ Lambda = 0.09, *η*_*p*_^2^ = 0.56); and (c) DO and pH (*p* = 0.21, Wilks’ Lambda = 0.24, *η*_*p*_^2^ = 0.52).

The results of the two-way RM-MANOVA indicated that the effect of climate scenario is only significant for DO among all other assessed physicochemical variables (Table 1). The effect of water level management was significant for all physicochemical variables except for TP and DO (Table 1). The greatest impact of water level management was for NH_4_-N and BOD_5_ and the smallest one for DO (Table 1). The interactive effect of water level management and climate scenario was found to be significant only for TSS, but not for any other examined variables (Table 1).

**Table 1:** Results of the two-way repeated-measures multivariate analysis of variance (RM-MANOVA) representing the effect of the main factors climate scenario, management (water level management), and their interactions (climate scenario × management) on the physicochemical variables

Variable (factor)	Climate scenario				Management				Climate scenario*management			
	*df*	*F*	*P*	*η*_*p*_^2^	*df*	*F*	*P*	*η*_*p*_^2^	*df*	*F*	*P*	*η*_*p*_^2^
TP	3	1.44	0.336	0.46	1	2.76	0.158	0.37	3	0.38	0.771	0.19
NH_4_-N	3	1.00	0.467	0.37	1	97.19	** < 0.001**	0.95	3	1.60	0.300	0.49
DOC	3	1.85	0.22	0.41	1	20.28	**0.002**	0.72	3	0.73	0.564	0.21
COD	3	1.45	0.30	0.35	1	47.38	** < 0.001**	0.86	3	2.12	0.176	0.44
BOD_5_	3	2.44	0.180	0.59	1	87.64	** < 0.001**	0.95	3	4.82	0.062	0.74
DO	3	4.82	**0.049**	0.71	1	0.65	0.451	0.10	3	1.95	0.224	0.49
TSS	3	2.52	0.13	0.49	1	47.65	** < 0.001**	0.86	3	4.52	**0.039**	0.63
pH	3	0.88	0.503	0.31	1	15.95	**0.007**	0.73	3	2.39	0.167	0.55

Partial Eta squared (*η*_*p*_^2^) denotes the effect size. Bold *p*-values indicate a statistical significance at *p* < 0.05

*TP* total phosphorous (mg/l), *NH*_*4*_*-N* ammonium-nitrogen (mg/l), *DOC* dissolved oxygen demand (mg/l), *COD* chemical oxygen demand (mg/l), *BOD*_*5*_ 5-day biochemical oxygen demand (mg/l), *DO* dissolved oxygen (mg/l), *TSS* total suspended solids (mg/l)

The results of the pairwise Bonferroni test showed that there are no statistically significant differences between the mean values of any physicochemical variables for different climate scenarios concerning both managed and unmanaged mesocosms, with the exception of TSS, which showed a significant difference between RCP 8.5 and CC for the unmanaged system (Fig. 2f). In terms of the mean values of TP and DO, no significant difference was identified between the managed and unmanaged systems at any level of climate scenario (Fig. 2a,d). A pairwise comparison showed significant differences between the COD and TSS mean values of managed and unmanaged systems under all future climate scenarios (Fig. 2g, f). However, for the current climate, there was no significant difference between the COD and TSS mean values of managed and unmanaged mesocosms (Fig. 2g, f).

The NH_4_-N mean values of unmanaged systems were significantly higher than the ones for the managed systems under all climate scenarios (Fig. 2c). Unexpectedly, only RCP 8.5 revealed a significant difference between the DOC mean value of the managed and unmanaged mesocosms, but not the other climate scenarios (Fig. 2e). The mean BOD_5_ concentrations of the unmanaged mesocosms under all climate scenarios were significantly higher than the managed ones, except under RCP 4.5 (Fig. 2h). For all climate scenarios, except for RCP 2.6, the mean pH levels in the unmanaged mesocosms were significantly higher than those for the managed ones (Fig. 2b).

**Fig. 3:**
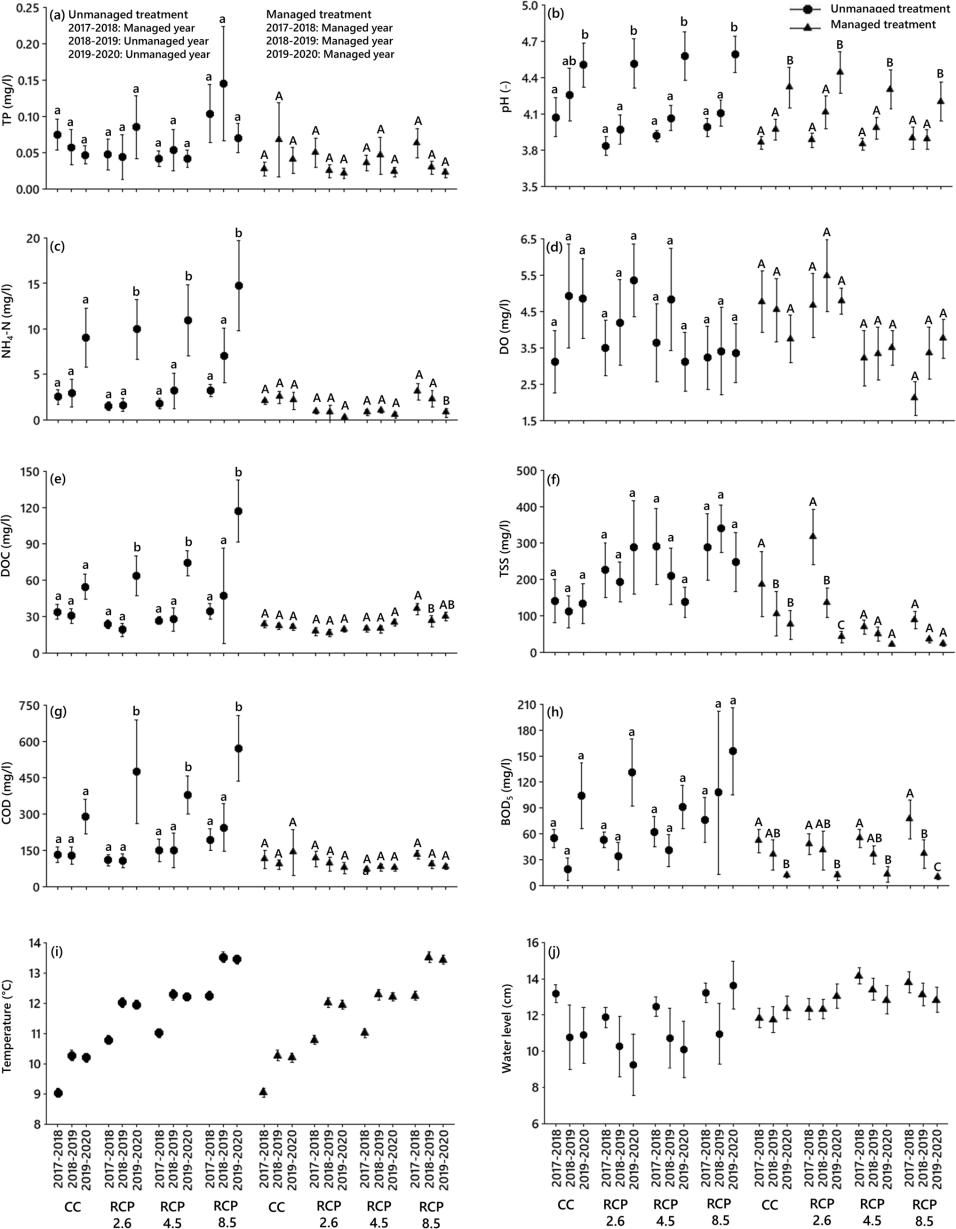
Trends of **a** total phosphorus (TP), **b** pH, **c** ammonium-nitrogen (NH_4_-N), **d** dissolved oxygen (DO), **e** dissolved organic carbon (DOC), **f** total suspended solids (TSS), **g** chemical oxygen demand (COD), **h** 5-day biochemical oxygen demand (BOD_5_), **i** temperature, and **j** water level for peatland mesocosms (unmanaged and managed treatment) under different climate scenarios (current climate (CC) and future representative concentration pathway (RCP) climate scenarios (RCP 2.6, RCP 4.5, and RCP 8.5) over a 3-year experimental period (2017–2020) simulating the real time for the period 2016–2019. During the first year of the experiment, all peatland mesocosms (4 mesocosms) were subject to water level management (2017–2018). In the second and third years of the experiment (2018–2020), two mesocosms were not managed (unmanaged treatment), but another two mesocosms were continued to be managed in terms of water level (managed treatment)

Figure 3 shows the trends of selected parameters for peatland mesocosms under different climate scenarios over a 3-year experimental period. The water quality deteriorated considerably in unmanaged treatment over time, where the water level was regulated during the first year of the experiment (2017–2018), but left unmanaged in subsequent years (2018–2020). For all climate scenarios, except for the current climate, the mean values of NH_4_-N, DOC, and COD as well as the concentrations of the mesocosms subject to unmanaged treatment for the period 2019–2020 were significantly higher compared to the periods 2017–2018 and 2018–2019 (Fig. 3c, e, g). However, there was no statistically significant difference between the periods 2017–2018 and 2018–2019 for these variables concerning all climate scenarios (Fig. 3c, e, g). The average TP, DO, TSS, and BOD_5_ values for unmanaged treatment did not differ significantly over the years (Fig. 3a, d, f, h). The pH was elevated in both managed and unmanaged treatments for all climate scenarios over time (Fig. 3b). Under all climate scenarios, the pH values of the unmanaged treatment for the period 2019–2020 were significantly higher than for the 2017–2018 and 2018–2019 periods, with the exception of the current climate (Fig. 3b).

The water quality remained relatively stable or improved for the managed treatment (throughout the whole experimental period) as most physicochemical variables decreased or remained relatively constant (Fig. 3). For example, there were no significant differences in the mean TP, DO, and COD concentrations for the managed treatment between the years of the experiment (Fig. 3a, d, g).

The mean DOC concentrations for the managed treatment under RCP 8.5 were significantly greater for the period 2017–2018 than for 2018–2019 (Fig. 3e). Furthermore, under RCP 8.5, the mean NH_4_-N concentrations for the managed treatment were significantly lower for the period 2019–2020 than for both 2017–2018 and 2018–2019 (Fig. 3c). For all climate scenarios, the mean BOD_5_ of the managed treatment for the period 2017–2018 was shown to be significantly higher than for 2019–2020 and for RCP 8.5. All annual data were significantly different from each other (Fig. 3h). The mean TSS of managed treatment for the period 2017–2018 was significantly higher than for both 2018–2019 and 2019–2020 under the current climate scenario. However, under RCP 2.6, all years were significantly different from one another. Moreover, there was no significant difference between the years under RCP 4.5 and RCP 8.5 (Fig. 3f).

## Discussion

The findings highlight the critical impact of water level management on the water quality of peatlands. In contrast, the effect of climate scenario on peatland water quality was not statistically significant over the period of this study. However, the authors expect that the effect of climate scenario on peatland water quality may become statistically significant in the longer term, as an increasing trend for some physicochemical variable concentrations for warmer climate scenarios has already been identified (Fig. 2; unmanaged treatment). Another reason why the climate scenario has not been identified as a statistically significant factor could be that future RCP climate scenarios for the studied region (southern Sweden characterized by continental and temperate climate) anticipate increases in both temperature and precipitation (Fig. S1b,d). As a result, the negative impact of higher evapotranspiration might have been mitigated by higher rates of precipitation under warmer climate scenarios in this study. However, the significance of climate change may be more pronounced in regions where decreased precipitation and increased temperature are recorded at the same time, causing water level drops for a longer period of time.

Water level management considerably improves the water quality of peatland outflow under each climate scenario by lowering the concentration of almost all physicochemical variables. This improvement was the greatest for ammonium (NH_4_-N) with reductions of up to 92% under RCP 2.6, followed by 5-day biochemical oxygen demand (BOD_5_) with decreases of up to 83% under RCP 8.5. However, the improvement for total phosphorus (TP) was not as great as in other variables, but still considerable reductions of up to 77% were recorded for the warmest climate scenario (RCP 8.5). Drought had the most detrimental impact on some chemical variables, increasing their concentrations in the unmanaged mesocosm outflows, consequently deteriorating water quality. However, it was shown that water level management could reduce the negative impact of drought and subsequent peat decomposition substantially.

The concentration of DOC in the unmanaged mesocosms increased along the climate scenario gradient from the coldest (current climate) to the warmest simulated climate (RCP 8.5). Although the impact of the climate scenarios was not found to be significant, there was an increasing trend from the coldest to the warmest climate scenario for most measurements. This suggests that higher temperatures and lower water levels could increase particularly aerobic microbial activity and solubility of some nutrients (Evans et al. 1999; Blodau 2002), accelerating peat mineralization (Gough et al. 2016). This commonly results in an increase in DOC leaching (Eimers et al. 2003; Clark et al. 2007; Porcal et al. 2009) over time. Menberu et al. (2017) noted that the drawdown of the water table in a boreal-rich fen by only 3 cm could increase the DOC production by 21.8%. The 2018 drought increased DOC concentrations in the unmanaged system over the summer and autumn, when rain washed out the unsaturated and aerated peat, resulting in high DOC concentrations that lasted until the following year.

Our findings are in accordance with those of Ritson et al. (2017) who found that mild droughts can result in an increase of about 40% DOC production from peat. However, our results contradicted some findings from other studies (Clark et al. 2012; Juckers and Watmough 2014), which reported a significant decline of DOC in response to the simulated drought. Drought, on the other hand, may have little influence on the amount of DOC produced by vegetation litter (Ritson et al. 2017). As a result, future concerns should focus on the impact of drought on peat decomposition, resulting in a release of recalcitrant DOC, which is difficult to be removed using conventional water treatment methods such as the coagulation and flocculation process (Ritson et al. 2017).

**Fig. 4:**
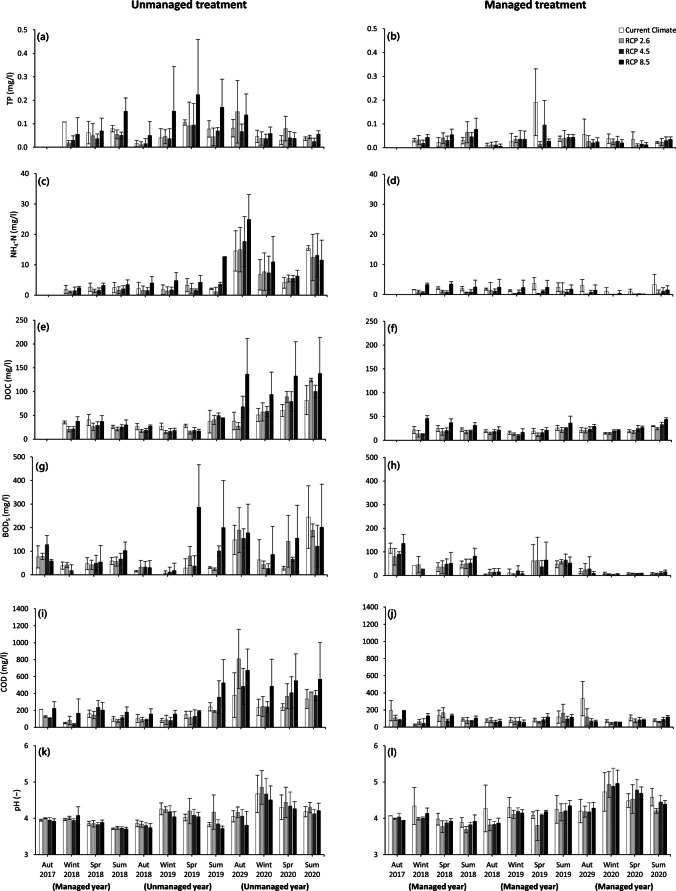
Seasonal averages for **a** total phosphorus (TP) regarding unmanaged treatment, **b** TP concerning managed treatment, **c** ammonium-nitrogen (NH_4_-N) regarding unmanaged treatment), **d** NH_4_-N concerning managed treatment, **e** dissolved organic carbon (DOC) regarding unmanaged treatment, **f** DOC concerning managed treatment, **g** 5-day biochemical oxygen demand (BOD_5_) regarding unmanaged treatment, **h** BOD_5_ concerning managed treatment, **i** chemical oxygen demand (COD) regarding unmanaged treatment, **j** COD concerning managed treatment, **k** pH regarding unmanaged treatment, and **l** pH concerning managed treatment under four climate scenarios (current climate (CC) and future RCP (representative concentration pathways) climate scenarios (RCP 2.6, RCP 4.5, and RCP 8.5)) for the peatland mesocosms over the period of the experiment from 2017 to 2020 (equivalent to real time between 2016 and 2019). During the first year of the experiment, all four peatland mesocosms were subject to water level management (autumn 2017–summer 2018). In the second and third years of the experiment (autumn 2018–summer 2020), two mesocosms were not managed (unmanaged treatment), but another two mesocosms were continued to be managed in terms of water level (managed treatment)

The managed mesocosms in this study had just a slight increase in DOC during the drought of summer 2018, indicating that water level management had a substantial impact, which reduced the concentration of DOC between three and four times in the managed mesocosms compared to the unmanaged system during the post-drought period from autumn 2019 until the end of summer 2020) (Fig. 4f) (Ritson et al. 2017). The observed increase in DOC concentrations during and after droughts is consistent with previous research (Worrall et al. 2006). Other studies that assessed the impact of rising water levels on DOC release in drained peatlands reported both an increase (Koskinen et al. 2011) and a decrease or no effect (Ramchunder et al. 2009; Armstrong et al. 2010; Wilson et al. 2011).

The BOD_5_ concentration in the unmanaged mesocosms responded to drought in the warmer climate scenarios (RCP 4.5 and RCP 8.5) earlier than those in the colder climate scenarios. This is due to the fact that, in warmer climate scenarios, higher temperatures for longer periods of time enhance microbial activity. This can result in a faster degradation of peat and vegetation litter and the subsequent release of more biodegradable organic matter in warmer compared to colder climate (Fenner and Freeman 2011). However, under the current climate scenario and RCP 2.6, biodegradable organic matter leached out from the unmanaged mesocosms with a delay during autumn, when temperatures decreased and the mesocosms received more rainwater. No trend was found for BOD_5_ of the unmanaged mesocosms under different climate scenarios during the growing season of 2020 due to the fact that BOD_5_ is also controlled by substrate quality in addition to environmental factors. Water level management of the managed mesocosms could significantly reduce the BOD_5_, and all managed mesocosms in all climate scenarios revealed a decrease between 7 and 17 times over the experimental period (Fig. 4g, h).

The concentration of COD decreased between two and seven times in the managed mesocosms compared to the unmanaged ones as a result of the significant effect of water level management (Fig. 4i, j). These findings are consistent with those of Saarinen et al. (2010) who reported a significant positive correlation between the catchment proportion percentage of a drained peatland and COD concentrations.

According to some findings, prediction of phosphorus response to environmental factors is not straightforward (Vasander et al. 2003; Urbanová et al. 2011); neither water level (Fölster et al. 2014) nor temperature (Munir et al. 2017) change is likely to be a significant driver for phosphorus release. In this study, during spring and summer, the release of phosphorus increased in the unmanaged mesocosms (Fig. 4a), most likely due to their highly readily soluble peat phosphorus concentration as a result of peat decomposition (Kaila et al. 2016). Moreover, managed mesocosms had lower phosphorus concentrations than unmanaged ones (Fig. 4b). Our findings are in line with what Munir et al. (2017) noted; the higher water levels decrease the phosphorus concentration in the outflow. They, however, observed a significant effect of the water level on phosphorus availability, but this was not the case in this study, although a decreasing trend of phosphorus concentration was found in this project, too (Fig. 4a, b). Other studies reported decreases (Lundin and Bergquist 1990), increases (Kløve et al. 2010), and no differences (Macrae et al. 2013) in terms of phosphorus with the water level drawdown. These discrepancies could be attributed primarily to the fact that nitrogen and phosphorus are limiting nutrients in ombrotrophic bogs, which have a high plant productivity (Idol et al. 2007; Iversen et al. 2010). Drawdown of water levels creates an aerobic zone, which promotes vascular vegetation expansion and growth (Munir et al. 2014), and their deeper root systems may absorb more phosphorus from the peat. As a result, while the production of phosphorus and nitrogen may rise with reduced water levels, plants can consume them at the same or even higher rate.

Warmer climate and lower moisture content, as seen during droughts and especially in warmer climate scenarios, can result in the top soil being well aerated. This is favorable for nitrification and the production of NO_3_. In our study, measurements of NO_3_ over the first 6 months revealed that NO_3_ has a very low concentration, which was lower than the quality standards for surface freshwater. However, NH_4_-N had a high concentration (Westbrook and Devito 2004). Although our peatland mesocosms had aerobic conditions in their top part, it was expected that nitrification lowers the NH_4_-N concentration, but presumably a low pH might have restricted nitrification activity (Bridgham et al. 2001), leading to the dominance of NH_4_-N (Wang et al. 2016).Munir et al. (2017) quantified the nutrient release from a Canadian bog in response to experimental warming and water table lowering. They noted that the NH_4_-N concentration within peat increased with water table depth and time. They also reported that warming treatment alone could not change the NH_4_-N concentration. These results are in line with this study in that sense that the impact of the climate scenario alone was not essential. However, the water level management had a strong effect that could result in a decrease of NH_4_-N concentration between 4 and 50 times in the managed mesocosms compared to the unmanaged ones during the post-drought period between autumn 2019 and the end of summer 2020 (Fig. 4c, d) (Frank et al. 2014; Laine et al. 2013). The decreasing trend of NH_4_-N as a result of water level management validates Lundin et al.’s (2017) findings indicating that rewetting can have a major impact on lowering the amount of NH_4_-N that lasts for a long time.

**Fig. 5:**
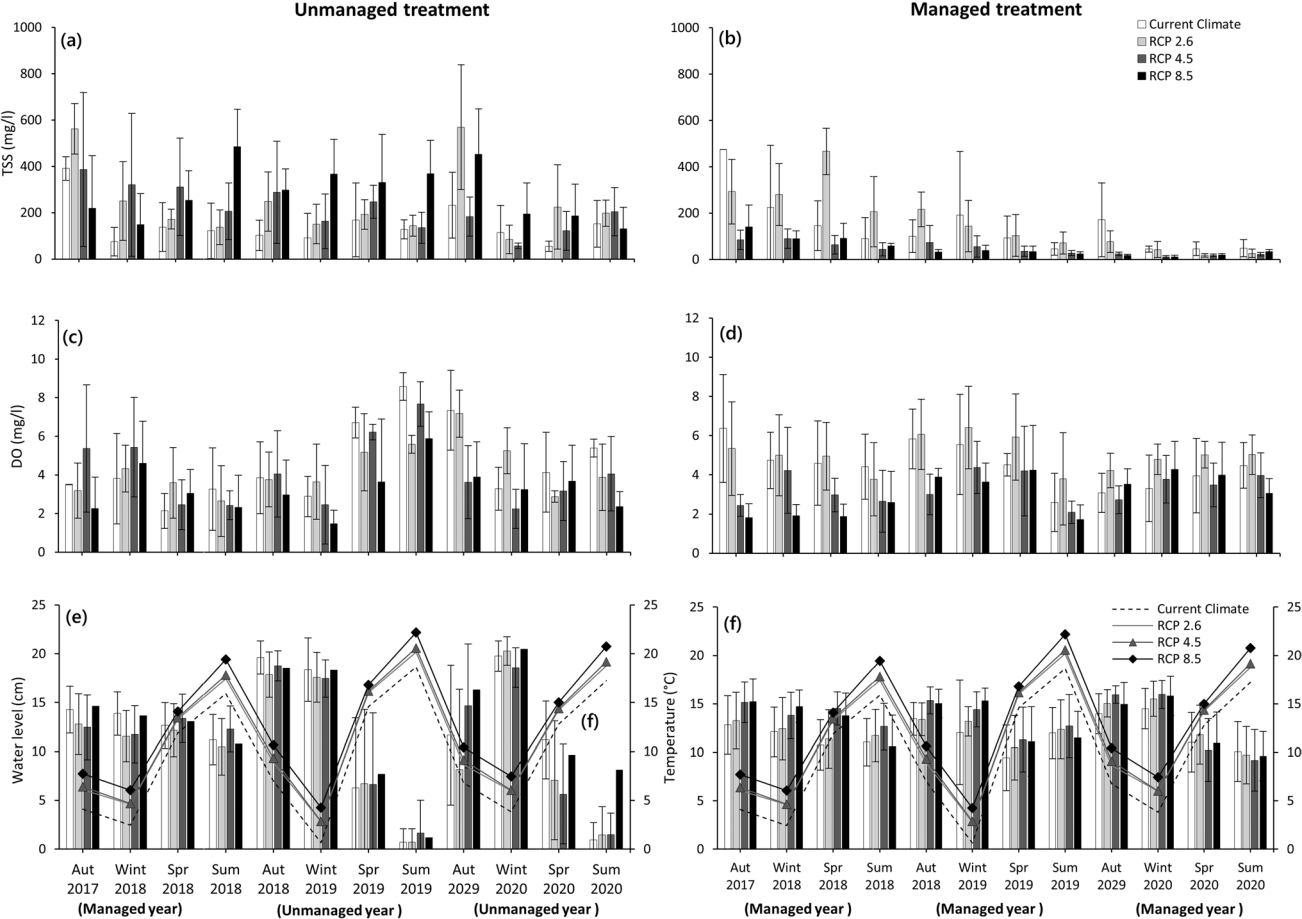
Seasonal averages for **a** total suspended solids (TSS) regarding unmanaged treatment, **b** TSS concerning managed treatment, **c** dissolved oxygen (DO) regarding unmanaged treatment, **d** DO concerning managed treatment, **e** water level, and **f** temperature under different climate scenarios for the peatland mesocosms under four climate scenarios (current climate (CC) and future representative concentration pathway (RCP) climate scenarios (RCP 2.6, RCP 4.5, and RCP 8.5)) for the peatland mesocosms over the period of the experiment from 2017 to 2020 (equivalent to real time between 2016 and 2019). During the first year of the experiment, all four peatland mesocosms were subject to water level management (autumn 2017–summer 2018). In the second and third years of the experiment (autumn 2018–summer2020), two mesocosms were not managed (unmanaged treatment), but the other two mesocosms were continued to be managed in terms of water level (managed treatment)

Lowering the water tables of peatlands increases the load of suspended solids in peatland outflows over the short term (Fig. 5a). This is of importance as in nature, peat particles (as part of TSS) can be transported to water bodies downstream, subsequently fall out of suspension and decompose, potentially causing oxygen depression, which has a negative impact on aquatic life. Furthermore, the decomposition of organic matter in the aquatic system might lead to an increase in atmospheric carbon dioxide levels (Davidson and Janssens 2006). In our study, the comparison of managed and unmanaged mesocosms revealed that water level management could reduce the TSS of the mesocosms for all climate scenarios; this effect was statistically significant for all future climate scenarios (Table 1; Fig. 5a, b). The efficiency of water level management was such that it could reduce TSS by between 60 and 90% in the managed mesocosms over 3 years (Fig. 5a,b). These findings support the outcomes of Martin-Ortega et al. (2014), who found strong evidence of rapid (less than 5 years) responses of suspended sediments to peatland re-wetting. While all of the managed mesocosms showed a constant decreasing pattern over time, the unmanaged mesocosm TSS trend was inconsistent among climate scenarios. This is due to the interaction impact of climate change and water level, which appeared to have a significant effect on TSS change over time. As a result of this effect, for colder temperature scenarios, the current climate scenario and RCP 2.6 showed a rising trend, whereas the warmer climate scenarios (RCP 4.5 and 8.5) were characterized by a declining trend. These results can be attributed to the dilution effect of rain, as the unmanaged mesocosms, which were subjected to warmer climate scenarios, experienced more rain during autumn 2020 than the unmanaged mesocosms that were exposed to colder climate scenarios.

Under all climate scenarios, both managed and unmanaged systems showed an increasing trend in pH over time (Fig. 3b). However, this increase was greater in the unmanaged system due to increased nutrient and labile carbon levels, which raised the pH (Fenner and Freeman 2011). In general, a higher pH of the peatland outflow can be expected in the face of climate change. However, this can be slightly lowered by water level management.

The findings revealed that water level control had a significant favorable impact on peatland water quality. Salimi and Scholz (2021) assumed but not confirmed this finding also for the period of 2017–2018, when all of the mesocosms were subject to water level regulation. During the first year (2017–2018), the impact of climate scenarios in terms of higher temperature was evident; e.g., the RCP 8.5 scenario showed the highest concentration of all physicochemical variables (Salimi and Scholz 2021). The increasing concentration trend for most physicochemical variables concerning the gradient of the four tested climate scenarios was observed in this study for the period of 2018–2020 for the unmanaged mesocosms, but not for the managed ones. This demonstrates the positive impact of water level regulation (particularly increasing the water level during a dry growing season) for warmer scenarios, which can reverse the adverse impact of higher temperatures. According to Salimi et al. (2021b), who investigated the impact of water level management on the carbon dioxide sink function of peatlands under different climate scenarios, climates with lower temperatures may require less intensive water level management. As a result, the findings of all our studies (this study; Salimi and Scholz, 2021; Salimi et al. 2021b) on peatland mesocosms showed the importance of water level management for the warmer climatic scenarios, which could improve both water quality and the carbon dioxide sink function of peatlands substantially.

## Conclusions and recommendations

The results of this study highlight the beneficial effect of water level management, which could significantly improve the water quality of peatland outflow under each climate scenario by decreasing the concentrations of most physicochemical variables. The observed increasing concentration trend in terms of physicochemical variables for unmanaged mesocosms along the gradient of the four climate scenarios indicates that higher temperatures of future climate scenarios have a greater impact on water quality of peatlands over a longer time. Furthermore, the impact of the climate scenario on peatland water quality outflow may have been mitigated in the study region (southern Sweden), as an increase in precipitation may have offset the detrimental impact of higher temperatures on peatland decomposition. For those regions, where both a declining trend in precipitation and an increasing trend in temperature are predicted along the gradient of future climatic scenarios, the detrimental impact of climate change on peatland water quality should be more pronounced.

This study showed that drought had an adverse effect on peatland water quality. The increases in nutrient concentrations following the drought were long-lasting, and it is possible that it will continue in future years. However, water level management showed a considerable effect on lowering the concentration of all water quality indicators including nutrients to levels up to 50 times.

Most efficient water level management in terms of lowering physicochemical parameter concentrations was found under RCP 8.5. In contrast, management in terms of water level adjustment was least effective under the current climate scenario indicating the necessity of water level management for the warmer climate scenarios.

Our findings highlight the need for water level management in stabilizing nutrient levels in peatland outflows to mitigate the negative consequences of global warming and drought, as any harmful effects are difficult to be reversed. Not only the negative consequences of climate change and drought on peatland can impair the water quality of receiving watercourses, but they can also increase the greenhouse gas emissions from peatland as well as receiving water bodies. Therefore, our findings stress the positive impact of water level management particularly during droughts that can protect ecosystem services such as clean water provision and climate change mitigation for peatlands and receiving watercourses at the same time.

Climate change is a long-term phenomenon and is characterized by a gradual increase in carbon dioxide concentrations of atmosphere. Therefore, a long-term experimental simulation of along with simulations of carbon dioxide increase for the future key climate scenarios is strongly recommended to obtain more accurate long-term results. The beneficial impact of water level regulation, particularly maintaining water levels during drought, has been clearly demonstrated in this study. However, the degree of water level regulation required for each climate scenario may differ. This should be explored further to conserve natural resources and provide more sustainable management guidelines.

## Supplementary Information

Below is the link to the electronic supplementary material.

Supplementary file1 (DOCX 26.8 KB)

Supplementary Fig. 1 (PNG 821 KB)

High Resolution Image (TIF 9.35 MB)

Supplementary Fig. 2 (PNG 3.41 MB)

High Resolution Image (TIF 42.7 MB)

## Data Availability

The data that support the findings of this study are available from the corresponding author upon reasonable request.
