# Supplementary material for: Importance of water level management for peatland outflow water quality in the face of climate change and drought
Source: Environ Sci Pollut Res Int. 2022 Jun 2;29(50):75455–70. doi: 10.1007/s11356-022-20614-2 (PMC9553818; doi:10.1007/s11356-022-20614-2)
Supplement: Supplementary file 1 — Supplementary file1 (DOCX 26.8 KB) [file 11356_2022_20614_MOESM1_ESM.docx]

**Table S1.** List of the regional climate models (RCM), which have been used for the simulation of future scenarios for RCP 2.6, 4.5 and 8.5.

| Model | Full name of the RCM model | RCP | Reference |
| --- | --- | --- | --- |
| CCCma-CanESM2 | Canadian Centre for Climate Modelling and Analysis- Canadian earth system model | 4.5 and 8.5 | Arora et al. (2011) |
| CNRM-CERFACS-CNRM-CM5 | Centre National de Recherches Météorologiques, France – Centre Europe´en De Recherche et de Formation Avance´e-Component Model 5 | 4.5 and 8.5 | (Voldoire et al., 2013) |
| CSIRO-QCCCE-CSIRO-Mk3-6-0 | Commonwealth Scientific and Industrial Research Organisation, Mark 3.6.0 | 4.5 and 8.5 | Jeffrey et al. (2013) |
| ICHEC-EC-EARTH | Irish Centre for High-end Computing k – European Community-Earth-system Model | 2.6, 4.5 and 8.5 | Hazeleger et al. (2012) |
| IPSL-IPSL-CM5A-MR | Institut Pierre Simon Laplace Climate Model 5A-Medium Resolution | 4.5 and 8.5 | (Hourdin et al., 2013)) |
| MIROC-MIROC5 | Model for Interdisciplinary Research on Climate- | 2.6, 4.5 and 8.5 | Watanabe et al. (2010) |
| MOHC-HadGEM2-ES | Met Office Hadley Centre-Hadley Centre Global Environment Model version 2 | 2.6, 4.5 and 8.5 | Collins et al. (2011) |
| MPI-M-MPI-ESM-LR | Max Planck Institute for Meteorology – Earth System Model – low resolution | 2.6, 4.5 and 8.5 | Giorgetta et al. (2013) |
| NCC-NorESM1-M | Norwegian Climate Centre – Norwegian Earth System Model, Intermediate Resolution | 2.6, 4.5 and 8.5 | Bentsen et al. (2013) |
| NOAA-GFDL-GFDL-ESM2M | National Oceanic Atmospheric Administration – Geophysical Fluid Dynamics Laboratory – Earth System Model | 4.5 and 8.5 | (Dunne et al., 2012) |

**References**

Arora VK, Scinocca JF, Boer GJ, Christian JR, Denman KL, Flato GM, Kharin VV, Lee WG, Merryfield WJ (2011) Carbon emission limits required to satisfy future representative concentration pathways of greenhouse gases. Geophys Res Lett https://doi.org/10.1029/2010GL046270

Bentsen M, Bethke I, Debernard JB, Iversen T, Kirkevåg A, Seland Ø, Drange H, Roelandt C, Seierstad IA, Hoose C, Kristjánsson JE (2013) The Norwegian Earth System Model, NorESM1-M – Part 1: Description and basic evaluation of the physical climate. Geosci Model Dev https://doi.org/10.5194/gmd-6-687-2013

Collins WJ, Bellouin N, Doutriaux-Boucher M, Gedney N, Halloran P, Hinton T, Hughes J, Jones CD, Joshi M, Liddicoat S, Martin G, O’Connor F, Rae J, Senior C, Sitch S, Totterdell I, Wiltshire A, Woodward S (2011) Development and evaluation of an Earth-System model – HadGEM2. Geosci Model Dev https://doi.org/10.5194/gmd-4-1051-2011

Dunne JP, John JG, Adcroft AJ, Griffies SM, Hallberg RW, Shevliakova E, Stouffer RJ, Cooke W, Dunne KA, Harrison MJ, Krasting JP, Malyshev SL, Milly PCD, Phillipps PJ, Sentman LT, Samuels BL, Spelman MJ, Winton M, Wittenberg AT, Zadeh N (2012) GFDL’s ESM2 global coupled climate-carbon earth system models. Part I: Physical formulation and baseline simulation characteristics. J Clim https://doi.org/10.1175/JCLI-D-11-00560.1

Giorgetta MA, Jungclaus J, Reick CH, Legutke S, Bader J, Böttinger M, Brovkin V, Crueger T, Esch M, Fieg K, Glushak K, Gayler V, Haak H, Hollweg H-D, Ilyina T, Kinne S, Kornblueh L, Matei D, Mauritsen T, Mikolajewicz U, Mueller W, Notz D, Pithan F, Raddatz T, Rast S, Redler R, Roeckner E, Schmidt H, Schnur R, Segschneider J, Six KD, Stockhause M, Timmreck C, Wegner J, Widmann H, Wieners K-H, Claussen M, Marotzke J, Stevens B (2013) Climate and carbon cycle changes from 1850 to 2100 in MPI-ESM simulations for the Coupled Model Intercomparison Project phase 5. J Adv Model Earth Syst https://doi.org/10.1002/jame.20038

Hazeleger W, Wang X, Severijns C, Ştefǎnescu S, Bintanja R, Sterl A, Wyser K, Semmler T, Yang S, van den Hurk B, van Noije T, van der Linden E, van der Wiel K (2012) EC-Earth V2.2: Description and validation of a new seamless earth system prediction model. Clim Dyn https://doi.org/10.1007/s00382-011-1228-5

Hourdin F, Foujols MA, Codron F, Guemas V, Dufresne JL, Bony S, Denvil S, Guez L, Lott F, Ghattas J, Braconnot P, Marti O, Meurdesoif Y, Bopp L (2013) Impact of the LMDZ atmospheric grid configuration on the climate and sensitivity of the IPSL-CM5A coupled model. Clim Dyn https://doi.org/10.1007/s00382-012-1411-3

Jeffrey S, Rotstayn L, Collier M, Dravitzki S, Hamalainen C, Moeseneder C, Wong K, Syktus J (2013) Australia’s CMIP5 submission using the CSIRO-Mk3.6 model. Aust Meteorol Oceanogr J https://doi.org/10.22499/2.6301.001

Voldoire A, Sanchez-Gomez E, Salas y Mélia D, Decharme B, Cassou C, Sénési S, Valcke S, Beau I, Alias A, Chevallier M, Déqué M, Deshayes J, Douville H, Fernandez E, Madec G, Maisonnave E, Moine MP, Planton S, Saint-Martin D, Szopa S, Tyteca S, Alkama R, Belamari S, Braun A, Coquart L, Chauvin F (2013) The CNRM-CM5.1 global climate model: Description and basic evaluation. Clim Dyn https://doi.org/10.1007/s00382-011-1259-y

Watanabe M, Suzuki T, O’Ishi R, Komuro Y, Watanabe S, Emori S, Takemura T, Chikira M, Ogura T, Sekiguchi M, Takata K, Yamazaki D, Yokohata T, Nozawa T, Hasumi H, Tatebe H, Kimoto M (2010) Improved climate simulation by MIROC5: Mean states, variability, and climate sensitivity. J Clim https://doi.org/10.1175/2010JCLI3679.1
